# Multidrug-resistant bacterial contamination of healthcare workers’ mobile phones, decontamination efficacy, and potential implications for hospital-acquired infections

**DOI:** 10.1038/s41598-026-64278-1

**Published:** 2026-08-08

**Authors:** Maha Mohammed Ali El-Dahshan, Hagar Said Mousa Nassar, Asmaa Yahia Sharfeldin, Maha Mostafa Hana, Alyaa Ibrahim Ahmed Abo Eliwa

**Affiliations:** 1 Department of Medical Microbiology & Immunology, Faculty of Medicine, Menoufia University, Shebin Elkom, Menoufia Egypt; 2 Department of Clinical Pathology, Faculty of Medicine, Menoufia University, Shebin Elkom, Menoufia Egypt; 3 Department of Public Health and Community Medicine, Faculty of Medicine, Menoufia University, Shebin Elkom, Menoufia Egypt; 4 Department of Tropical Medicine, Faculty of Medicine, Menoufia University, Shebin Elkom, Menoufia Egypt

**Keywords:** Mobile phone contamination, Healthcare workers, Multidrug resistance, Antimicrobial resistance, ESBL, Carbapenemase-producing organisms, Intensive care units, Healthcare-associated infections, Diseases, Health care, Medical research, Microbiology

## Abstract

Mobile phones are indispensable clinical tools for healthcare workers (HCWs), yet remain largely unaddressed within infection prevention and control (IPC) frameworks. Repeated handling during patient care combined with inconsistent disinfection may allow these devices to serve as multidrug-resistant (MDR) organism reservoirs contributing to healthcare-associated infections (HAIs). A hospital-based cross-sectional analytical study with an embedded intervention and post-intervention follow-up was conducted at Menoufia University Hospitals, Egypt (2025–2026). Swabs from 300 HCWs’ mobile phones across eight clinical areas were processed microbiologically and confirmed by Vitek 2. Antimicrobial susceptibility testing followed the Clinical and Laboratory Standards Institute (CLSI) 2025 guidelines. Bacterial burden was quantified by colony-forming units (CFU; low < 50, moderate 50–200, high > 200). Extended-spectrum β-lactamase (ESBL) and carbapenemase production were phenotypically confirmed, then genotypically characterized by multiplex PCR (blaTEM, blaSHV, blaCTX-M, blaOXA-48, blaNDM, blaIMP, blaVIM, blaKPC). Four decontamination methods were evaluated, and ward-level HAI rates correlated with MDR prevalence. Overall, 270/300 (90.0%) phones were contaminated, and 179/300 (59.6%) carried MDR organisms, with the highest burden in the intensive care unit (ICU). Coagulase-negative staphylococci (CoNS; 29.4%) and S. aureus (25.4%), including methicillin-resistant strains (MRSA), predominated. Among Gram-negative isolates (n = 169), ESBL production was detected in 24.9% and carbapenemase in 13.0%; blaTEM (13.6%) and blaOXA-48 (4.7%) were most prevalent, respectively. Sodium hypochlorite–impregnated wipes achieved the greatest bacterial load reduction (96.5%). Only 9.0% of HCWs reported daily phone disinfection; phone use during invasive procedures was the strongest MDR risk factor (adjusted odds ratio [AOR] 2.9; p < 0.001). At the ward level, MDR prevalence was strongly correlated with HAI rates (r = 0.986; p < 0.001). Post-intervention reductions in contamination (90.0%→78.0%), MDR carriage (59.6%→45.0%), and mean CFU (190.1 ± 80.3→95.5 ± 55.2) were all significant (p < 0.001). HCWs’ mobile phones are potential reservoirs of MDR organisms, including clinically important ESKAPE-associated pathogens. Integrating standardized phone hygiene into IPC programs, supported by evidence-based decontamination and molecular surveillance, is warranted, particularly in high-acuity clinical settings.

**Supplementary Information:**

The online version contains supplementary material available at 10.1038/s41598-026-64278-1.

## Introduction

Healthcare-associated infections (HAIs) impose a sustained global burden, contributing substantially to morbidity, prolonged hospitalization, increased healthcare expenditure, and preventable mortality. The World Health Organization (WHO) has designated HAIs a major public health priority, particularly in resource-limited environments where surveillance capacity and IPC infrastructure are inconsistent^1,2^. This challenge is compounded by the global rise of multidrug resistance (MDR), which restricts therapeutic options and worsens clinical outcomes^3^.

In high-acuity settings such as intensive care units (ICUs), the convergence of vulnerable patients, invasive devices, repeated antimicrobial exposure, and intense HCW–patient contact creates optimal conditions for acquisition and dissemination of resistant microorganisms. Transmission extends beyond direct contact to include indirect spread via contaminated fomites and high-touch surfaces. Although IPC programs traditionally prioritize hand hygiene, environmental decontamination, and device sterilization, increasing attention has turned to personal devices that exist outside standardized IPC frameworks^4,5^.

Mobile phones are indispensable in contemporary clinical practice, facilitating communication, electronic medical record access, and multidisciplinary care coordination. Despite their operational value, they are high-touch devices that are repeatedly handled during patient care, transported across clinical and non-clinical settings, and rarely subjected to standardized disinfection, rendering them biologically plausible reservoirs for pathogenic and antimicrobial-resistant microorganisms^4–6^. Multiple studies have reported high contamination rates of HCWs’ phones, with recovery of clinically relevant organisms including *Staphylococcus aureus*, coagulase-negative staphylococci (CoNS), *Klebsiella pneumoniae*, *Escherichia coli*, *Pseudomonas aeruginosa*, and *Acinetobacter baumannii*^1,2,7^. Detection of MDR and ESKAPE pathogens on mobile devices is particularly concerning given their strong implication in HAIs and enhanced environmental persistence^3,8^.

Behavioural practices further amplify contamination risk. Phone use during patient care, handling during invasive procedures, restroom use, device sharing, and inconsistent hand hygiene create repeated opportunities for microbial transfer^9–11^. Molecular surveillance, encompassing detection of extended-spectrum β-lactamase (ESBL) genes (*bla*TEM, *bla*SHV, *bla*CTX-M) and carbapenemase genes (*bla*KPC, *bla*NDM, *bla*OXA-48, *bla*IMP, *bla*VIM), provides mechanistic insight into resistance patterns and strengthens epidemiological interpretation^12,13^. Quantitative CFU estimation enables stratification of contamination severity and better approximates transmission risk gradients^14^.

Despite accumulating evidence, few studies integrate quantitative bacterial load assessment, molecular resistance profiling, behavioural risk analysis, ward-level gradients, and post-intervention reassessment within a unified analytical framework. This study was therefore designed to comprehensively evaluate: (i) contamination prevalence and severity; (ii) antimicrobial resistance patterns and molecular determinants; (iii) associated behavioural and occupational risk factors; (iv) decontamination method efficacy; and (v) the relationship between ward-level MDR burden and HAI rates, with short-term follow-up to assess the impact of a structured educational phone-hygiene intervention.

## Materials and methods

### Study design and setting

This hospital-based analytical study was conducted at Menoufia University Hospitals, Egypt, between 2025 and 2026, and combined a cross-sectional baseline assessment with a prospective interventional component and paired, before-and-after follow-up reassessment. The baseline sampling, the structured educational intervention, and the follow-up evaluation were carried out by a dedicated, trained infection-control team applying standardized procedures consistently across the study period, so that the design extended beyond a single cross-sectional snapshot and captured change over time in response to a defined intervention. This tertiary-care teaching complex provides comprehensive services across ICUs, burn unit, surgical, haematology/oncology, paediatric, medical, obstetrics/gynaecology, and general wards, operating under IPC policies aligned with national and international guidelines^5^. All procedures involving human participants (HCW questionnaire and phone sampling) were performed in accordance with the ethical standards of the institutional Research Ethics Committee and the 1964 Declaration of Helsinki and its later amendments.

### Study population and sampling

HCWs (physicians, nurses, and laboratory technicians) who routinely used personal mobile phones during clinical duties were eligible. Inclusion required active patient care engagement during the study period; HCWs who declined or did not carry phones on duty were excluded. A stratified sampling approach ensured proportional representation across clinical areas and professional categories^2^. A total of 300 phones were sampled (one per participant). Sample size was determined using OpenEpi, based on a reported contamination prevalence of 93%^1^, with 80% power and 95% confidence level.

### Data collection and behavioural assessment

A structured, pretested questionnaire captured demographic characteristics, professional role, clinical experience, educational level, IPC training receipt (past two years), daily phone use duration, handling during invasive procedures, restroom use, call answering during patient care, device sharing, and perceived awareness of contamination risk^11^. Phone-related characteristics (device type, protective cover, screen protector) were documented as potential risk factors. Self-reported data were complemented by anonymous observational spot-checks within participating wards, aggregated at ward level.

### Microbiological sampling and processing

Sterile cotton swabs moistened with sterile normal saline were used to sample four predefined phone surfaces, touchscreen/keypad, lateral edges, earpiece, and posterior surface, using rotational movements. Swabs were transported to the microbiology laboratory within one hour and inoculated onto blood agar and MacConkey agar, followed by aerobic incubation at 37 °C for 24–48 h^5^. Preliminary identification employed Gram staining and standard biochemical tests. All isolates were confirmed by Vitek 2 (bioMérieux, France) using appropriate GP/GN identification cards^7^.

### Quantification of bacterial load

CFU counts recovered per phone were categorized as low (< 50 CFU), moderate (50–200 CFU), or high (> 200 CFU) contamination severity^6^. For quantification, each swab was eluted by vortexing in 1 mL of sterile normal saline for 30 s to release adherent organisms, and a fixed 100 µL aliquot of the eluate was inoculated onto blood agar using the spread-plate technique. After aerobic incubation at 37 °C for 24–48 h, discrete colonies were enumerated on plates yielding a countable range (30–300 colonies), and the total CFU per phone was back-calculated from the plated volume and the elution factor. Plating was performed in duplicate and the mean count was used, with the same operator, media lot, and counting criteria applied at baseline and at follow-up to ensure comparability. Categories were used to compare severity across wards and evaluate post-intervention changes.

### Antimicrobial susceptibility testing

Susceptibility testing was performed by Vitek 2 with minimum inhibitory concentration (MIC) interpretation per the Clinical and Laboratory Standards Institute (CLSI) 2025 guidelines^15^. The antimicrobial panel included: penicillin, oxacillin, erythromycin, clindamycin, trimethoprim–sulfamethoxazole (TMP-SMX), vancomycin, linezolid, daptomycin, amoxicillin–clavulanate, piperacillin–tazobactam, ceftazidime, cefepime, aztreonam, ciprofloxacin, tetracycline, gentamicin, imipenem, meropenem, and colistin. Methicillin resistance was determined by oxacillin and cefoxitin MIC results. MDR was defined as resistance to ≥ 1 agent in ≥ 3 antimicrobial classes^3^. Quality control of susceptibility testing was ensured in each run using the reference strains *Escherichia coli* ATCC 25,922, *Staphylococcus aureus* ATCC 25,923, and *Pseudomonas aeruginosa* ATCC 27,853.

### Phenotypic ESBL and carbapenemase detection

ESBL screening employed third-generation cephalosporins (cefotaxime, ceftriaxone, ceftazidime); isolates with reduced susceptibility to ≥ 1 agent underwent confirmatory double-disk synergy testing per CLSI recommendations^15^. Carbapenemase screening was based on imipenem and meropenem results, with phenotypic confirmation by the modified carbapenem inactivation method (mCIM), interpreted per CLSI criteria^16^. *K. pneumoniae* ATCC 700,603 and *E. coli* ATCC 25,922 were included as ESBL-positive and ESBL-negative controls, respectively, and *K. pneumoniae* ATCC BAA-1705 and ATCC BAA-1706 as carbapenemase-positive and carbapenemase-negative controls for the mCIM assay.

### Molecular detection of resistance genes

Genomic DNA was extracted from confirmed Gram-negative isolates using a commercially available kit. Multiplex polymerase chain reaction (PCR) assays detected ESBL genes (*bla*TEM, *bla*SHV, *bla*CTX-M) and carbapenemase genes (*bla*IMP, *bla*VIM, *bla*NDM, *bla*KPC, *bla*OXA-48). Amplified products were resolved by agarose gel electrophoresis and visualized under UV illumination. Appropriate positive and negative controls were included in each run^13^. Primer sequences were adopted from previously validated multiplex PCR protocols^17^, as detailed in **Table ****S1****.**

### Evaluation of decontamination methods

Four decontamination approaches were evaluated under controlled conditions: sodium hypochlorite–impregnated wipes, 70% isopropyl alcohol, quaternary ammonium compound disinfectant, and electronic-safe wipes. Phones were randomly allocated to one of four groups (*n* = 75 per group). Swabs were obtained immediately before and five minutes after disinfectant application; post-disinfection swabs were collected into neutralizing broth (Dey–Engley or lecithin–polysorbate) to inactivate residual disinfectant before plating, and the CFU percentage reduction was calculated^10^.

### Healthcare-associated infection data

Ward-specific HAI rates were obtained from hospital infection control surveillance records and expressed as HAIs per 1,000 patient-days. These were correlated with ward-level MDR prevalence on mobile phones, consistent with environmental transmission modelling frameworks^14^.

### Post-intervention follow-up

A structured educational intervention was implemented in participating wards, encompassing a session on contamination risks, demonstration of appropriate disinfection techniques, and reinforcement of daily phone-cleaning recommendations^9^. The intervention was delivered by trained infection-control personnel as standardized small-group sessions of approximately 20 min, using a fixed slide set and a live demonstration of phone decontamination. The recommended procedure specified wiping all phone surfaces with a sodium hypochlorite–impregnated or 70% isopropyl alcohol wipe, observing the manufacturer’s contact time, at least once per shift and after any contact with patients or the clinical environment. The same content and demonstrator were used across all wards to maintain consistency, and participants were not informed of the exact date of repeat sampling in order to limit reactive (Hawthorne) behaviour. Two weeks post-implementation, repeat swab sampling was performed from the same phones using identical standardized procedures.

### Statistical analysis

Data were analysed using IBM SPSS (version 27) and the epiCalc program. Qualitative variables were presented as numbers and percentages and analysed by Chi-square test (χ²). Quantitative variables were expressed as mean ± standard deviation. Pearson’s correlation coefficient assessed relationships between continuous normally distributed variables. Paired analyses (paired t-test and McNemar test) evaluated post-intervention changes. Independent predictors of MDR contamination were identified by binary logistic regression. Candidate variables were first screened in univariable analysis, and those with *p* < 0.20 or established biological plausibility were entered into the multivariable model; the final model retained working in an ICU/critical-care setting, phone use during invasive procedures, restroom phone use, and daily phone disinfection. Multicollinearity was assessed using variance inflation factors, and overall model fit was evaluated with the Hosmer–Lemeshow goodness-of-fit test^18^, with results reported as adjusted odds ratios with 95% confidence intervals. The ward-level correlation between MDR prevalence and HAI rate was pre-specified as an exploratory analysis and was not adjusted for ward-level confounders. A two-tailed p-value ≤ 0.05 was considered statistically significant.

## Results

### Participant characteristics

Of 300 HCWs enrolled, 60.0% were female (mean age 34.8 ± 8.6 years). Nurses constituted the largest occupational category (46.7%), followed by physicians (30.0%) and laboratory technicians (23.3%). The ICU (20.0%) and surgical ward (18.3%) were the most represented clinical areas. Most participants held a bachelor’s degree (70.0%) and 53.3% had received IPC training within the previous two years. Half (50.0%) used their phones for 2–5 h daily; only 9.0% reported daily phone cleaning, while 54.3% cleaned their phones rarely or never (Table 1).

**Table 1:** Demographic and professional characteristics of participating healthcare workers (*n* = 300).

Variable	Category	*n*	%
Gender	Male	120	40.0
	Female	180	60.0
Age group (years)	< 30	95	31.7
	30–< 40	110	36.7
	40–49	65	21.7
	≥ 50	30	10.0
	Mean age ± SD	34.8 ± 8.6	—
Profession	Physicians	90	30.0
	Nurses	140	46.7
	Laboratory technicians	70	23.3
Years of experience	< 5 years	105	35.0
	5–10 years	95	31.7
	> 10 years	100	33.3
Clinical work area	Intensive care unit	60	20.0
	Burn unit	40	13.3
	Surgical ward	55	18.3
	Haematology/Oncology	35	11.7
	Paediatric ward	40	13.3
	Medical ward	30	10.0
	Obstetrics/Gynaecology	20	6.7
	General ward	20	6.7
Educational level	Bachelor’s degree	210	70.0
	Postgraduate degree	90	30.0
IPC training (past 2 years)	Yes	160	53.3
	No	140	46.7
Average daily phone use at work	< 2 h	75	25.0
	2–5 h	150	50.0
	> 5 h	75	25.0
Frequency of phone cleaning	Daily	27	9.0
	Weekly	110	36.7
	Rarely/Never	163	54.3

IPC: infection prevention and control; SD: standard deviation. Values are number (percentage) unless otherwise stated. For the mean age row, the third column reports mean ± SD.

### Contamination prevalence and severity by clinical area

Overall phone contamination was 90.0% (270/300), exceeding 80% in every ward. The ICU demonstrated the highest contamination rate (98.3%) and MDR prevalence (75.0%), with significantly elevated MDR odds versus the general ward (OR 4.5; 95% CI 1.6–13.1; *p* = 0.004). The burn unit similarly showed 97.5% contamination with 70.0% MDR carriage (OR 3.5; 95% CI 1.1–10.7; *p* = 0.025). High-level contamination (> 200 CFU) was most frequent in the ICU (48.3%) and burn unit (47.5%), confirming a clear gradient of microbial burden across clinical risk settings (Table 2).

**Table 2:** Phone contamination prevalence, multidrug-resistant carriage, and bacterial load severity by clinical area (*n* = 300).

Clinical Area	Phones (*n*)	Contaminated *n* (%)	MDR *n* (%)	OR (95% CI)†	*p*-value	Low CFU < 50 *n* (%)	Moderate CFU 50–200 *n* (%)	High CFU > 200 *n* (%)
Intensive care unit	60	59 (98.3)	45 (75.0)	4.5 (1.6–13.1)	0.004	9 (15.0)	21 (35.0)	29 (48.3)
Burn unit	40	39 (97.5)	28 (70.0)	3.5 (1.1–10.7)	0.025	7 (17.5)	13 (32.5)	19 (47.5)
Surgical ward	55	51 (92.7)	34 (61.8)	2.4 (0.9–6.9)	0.092	14 (25.5)	23 (41.8)	14 (25.5)
Haematology/Oncology	35	30 (85.7)	20 (57.1)	2.0 (0.7–6.1)	0.221	8 (22.9)	14 (40.0)	8 (22.9)
Paediatric ward	40	35 (87.5)	21 (52.5)	1.7 (0.6–4.9)	0.361	12 (30.0)	15 (37.5)	8 (20.0)
Medical ward	30	24 (80.0)	14 (46.7)	1.3 (0.4–4.1)	0.642	10 (33.3)	9 (30.0)	5 (16.7)
Obstetrics/Gynaecology	20	16 (80.0)	9 (45.0)	1.2 (0.4–4.3)	0.749	7 (35.0)	6 (30.0)	3 (15.0)
General ward (ref.)	20	16 (80.0)	8 (40.0)	—	—	9 (45.0)	5 (25.0)	2 (10.0)
**Total**	**300**	**270 (90.0)**	**179 (59.6)**	**—**	**—**	**—**	**—**	**—**

MDR: multidrug resistance (resistance to ≥ 1 agent in ≥ 3 antimicrobial classes); CFU: colony-forming units; OR: odds ratio; CI: confidence interval. †OR for MDR contamination versus the general ward (reference category). p ≤ 0.05 (Chi-square test).

### Bacterial isolate distribution and MDR burden

A total of 374 bacterial isolates were recovered. CoNS were most prevalent (29.4%, 110/374; 48.2% MDR), followed by *S. aureus* (25.4%, 95/374; 57.9% MDR). Among Gram-negative organisms, *K. pneumoniae* accounted for 16.0% (60/374; 61.7% MDR), *E. coli* for 12.0% (45/374; 55.6% MDR), *P. aeruginosa* for 9.4% (35/374; 68.6% MDR), and *A. baumannii* for 7.8% (29/374; 72.4% MDR). Overall, 57.5% (215/374) of all isolates were MDR (Table 3).

**Table 3:** Distribution of bacterial isolates recovered from healthcare workers’ mobile phones and multidrug-resistance burden by species (*n* = 374 isolates).

Bacterial Species	Total Isolates (*n*)	% of All Isolates	MDR (*n*)	MDR within Species (%)
Coagulase-negative staphylococci (CoNS)	110	29.4	53	48.2
*Staphylococcus aureus* (including MRSA)	95	25.4	55	57.9
*Klebsiella pneumoniae*	60	16.0	37	61.7
*Escherichia coli*	45	12.0	25	55.6
*Pseudomonas aeruginosa*	35	9.4	24	68.6
*Acinetobacter baumannii*	29	7.8	21	72.4
**Total**	**374**	**100.0**	**215**	**57.5**

MDR: multidrug resistance; MRSA: methicillin-resistant Staphylococcus aureus. MDR within species (%): proportion of isolates within each species classified as multidrug-resistant.

### Antimicrobial resistance profiles

Penicillin resistance was extremely high among Gram-positive organisms (*S. aureus* 95%; CoNS 90%), with oxacillin resistance at 58% and 65%, respectively. No vancomycin resistance was detected (0%), and linezolid resistance remained minimal (2–3%). Among Gram-negatives, carbapenem resistance was most marked in *A. baumannii* (imipenem 30%; meropenem 32%) and *P. aeruginosa* (imipenem 25%; meropenem 26%), with lower but clinically significant rates in *K. pneumoniae* (15–18%) and *E. coli* (13–15%). TMP-SMX resistance exceeded 60% in non-fermenters, markedly restricting therapeutic options (Table S2).

### Phenotypic ESBL and carbapenemase detection

Among 169 Gram-negative isolates, ESBL production was identified in 24.9% (42/169) and carbapenemase production in 13.0% (22/169). Phenotypic co-production was observed in 5.9% (10/169). ESBL prevalence was highest in *E. coli* (35.6%), followed by *A. baumannii* (24.1%), *P. aeruginosa* (22.9%), and *K. pneumoniae* (18.3%). Carbapenemase production was most prominent in *A. baumannii* (31.0%) and *P. aeruginosa* (20.0%) (Table 4).

**Table 4:** Phenotypic detection of extended-spectrum β-lactamase and carbapenemase production among Gram-negative isolates (*n* = 169).

Organism	Total Isolates (*n*)	ESBL Producer *n* (%)	Carbapenemase Producer *n* (%)	ESBL + Carbapenemase Co-producer *n* (%)
*Klebsiella pneumoniae*	60	11 (18.3)	2 (3.3)	1 (1.7)
*Escherichia coli*	45	16 (35.6)	4 (8.9)	2 (4.4)
*Pseudomonas aeruginosa*	35	8 (22.9)	7 (20.0)	3 (8.6)
*Acinetobacter baumannii*	29	7 (24.1)	9 (31.0)	4 (13.8)
**Total**	**169**	**42 (24.9)**	**22 (13.0)**	**10 (5.9)**

ESBL: extended-spectrum β-lactamase (confirmed by double-disk synergy test); carbapenemase confirmed by the modified carbapenem inactivation method (mCIM). Co-producers are counted within both the ESBL and carbapenemase columns. Percentages are calculated per species.

### Molecular resistance gene distribution

**Fig. 1:**
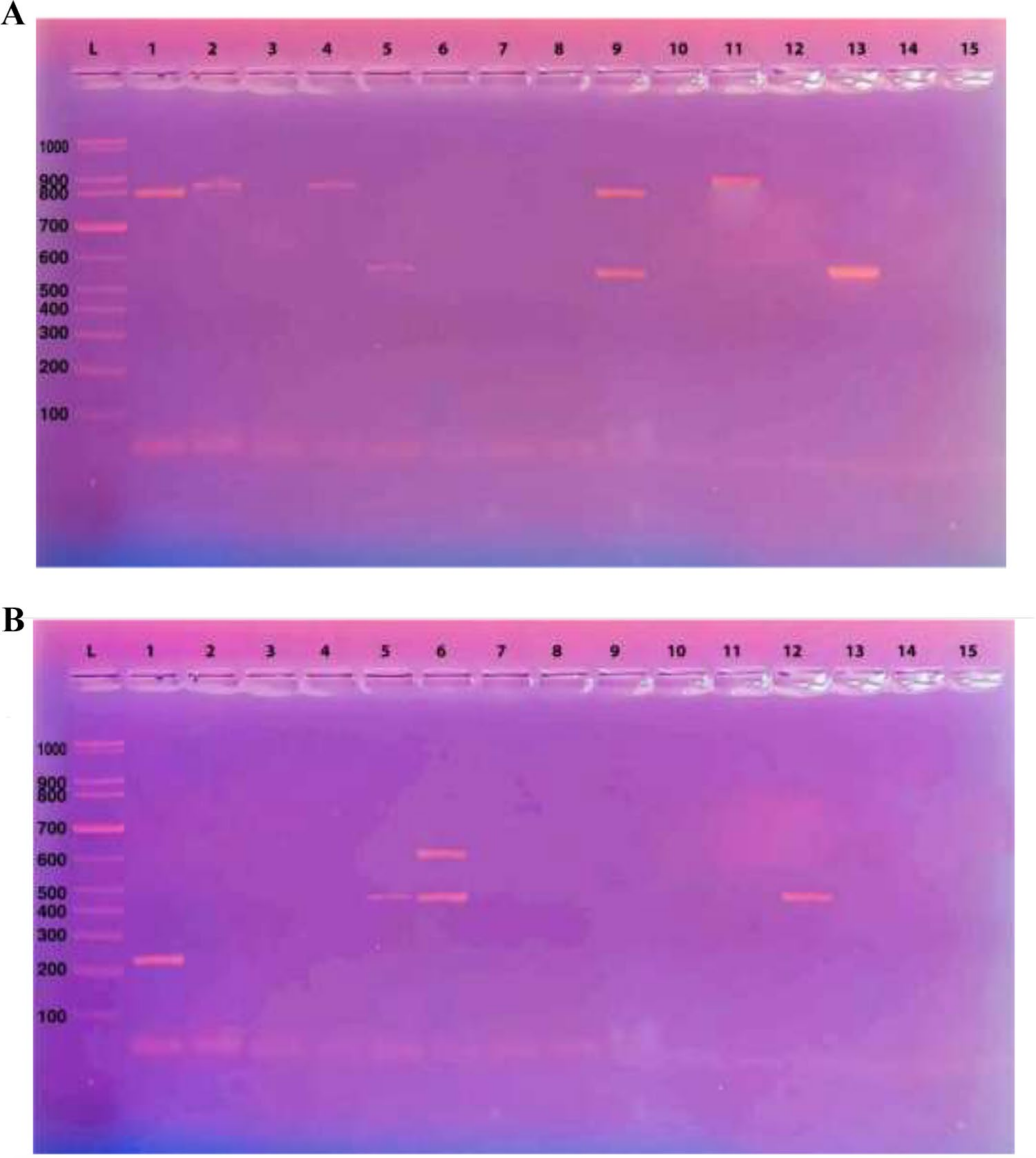
Molecular detection of resistance genes (ESBLs and carbapenemases). (**A**) PCR Gel Electrophoresis of the amplified product of ESBL genes *blaTEM*, *blaSHV*, and *blaCTX-M* genes. Lane L: DNA Molecular Size ladder (100−1000 bp). Lanes 1, and 9: Positive for *blaTEM *with a band size of 800bp. Lanes 2,4, and 11: Positive for* blaSHV* with a band size of 843 bp. Lanes 5, 9, and 13: positive for* blaCTX-M*. (**B**) PCR Gel Electrophoresis of the amplified product of carbapenemase genes *blaOXA-48*, *blaNDM*, and *blaIMP* genes. Lane L: DNA Molecular Size ladder (100−1000 bp). Lane 1: Positive for *blaIMP* with a band size of 232 bp. Lanes 5, 6 and 12: Positive for* blaOXA-48* with a band size of 438 bp. Lanes 6: positive for *blaNDM*.

The amplified products of ESBL and carbapenemase genes detected by multiplex PCR are shown in Fig. 1 (A and B). Molecular analysis demonstrated that 24.9% (42/169) of Gram-negative isolates carried ≥ 1 ESBL gene. *bla*TEM was most prevalent (13.6%, 23/169), followed by *bla*SHV (7.1%) and *bla*CTX-M (4.7%). Carbapenemase genes were detected in 7.1% (12/169), predominantly *bla*OXA-48 (4.7%), followed by *bla*NDM (1.8%) and *bla*IMP (0.6%); neither *bla*VIM nor *bla*KPC was identified. ESBL + carbapenemase co-genotypes were present in 5.9% (10/169) (Table 5; Fig. 1).

**Table 5:** Molecular distribution of extended-spectrum β-lactamase and carbapenemase resistance genes among Gram-negative isolates (*n* = 169).

Molecular Finding	No. of Isolates	% (*n* = 169)
**Any ESBL gene detected**	42	24.9
*blaTEM*	23	13.6
*blaSHV*	12	7.1
*blaCTX-M*	8	4.7
≥ 2 ESBL genes detected	1	0.6
**Any carbapenemase gene detected**	12	7.1
*blaOXA-48*	8	4.7
*blaNDM*	3	1.8
*blaIMP*	1	0.6
*blaVIM*	0	0.0
*blaKPC*	0	0.0
≥ 2 carbapenemase genes detected	1	0.6
ESBL-only genotype	32	18.9
Carbapenemase-only genotype	2	1.2
ESBL + carbapenemase genotype	10	5.9
**No target resistance gene detected**	125	74.0

PCR targets — ESBL genes: blaTEM, blaSHV, blaCTX-M; carbapenemase genes: blaOXA-48, blaNDM, blaIMP, blaVIM, blaKPC. Percentages are based on all Gram-negative isolates (n = 169). Isolates carrying both gene classes are counted in both categories.

### Phenotype–genotype diagnostic correlation

Phenotypic–molecular agreement for ESBL detection was perfect (sensitivity, specificity, PPV, NPV, and accuracy all 100%; κ = 1.00; *p* < 0.001). For carbapenemase detection, phenotypic methods identified more positive isolates than PCR (sensitivity 54.5%; specificity, PPV 100%; NPV 93.6%; accuracy 94.1%; κ = 0.67; *p* < 0.001), reflecting resistance mechanisms not targeted by the molecular panel (Table S3).

### Decontamination efficacy

All four methods produced statistically significant bacterial load reductions. Sodium hypochlorite–impregnated wipes were most efficacious (96.5%; mean CFU from 228.3 ± 40.1 to 8.3 ± 4.2; *p* < 0.001). 70% isopropyl alcohol achieved a comparable reduction (94.8%; *p* < 0.001). Quaternary ammonium compound produced 92.0% reduction (*p* < 0.001). Electronic-safe wipes were least effective but still significant (81.8%; *p* < 0.01) (Table 6).

**Table 6:** Efficacy of four decontamination methods in reducing phone bacterial load (*n* = 300; 75 phones per group).

Disinfection Method	Phones (*n*)	CFU Before Mean ± SD	CFU After Mean ± SD	Reduction (%)	*p*-value†
Sodium hypochlorite–impregnated wipes	75	228.3 ± 40.1	8.3 ± 4.2	96.5	< 0.001
70% isopropyl alcohol	75	230.5 ± 45.4	12.7 ± 5.5	94.8	< 0.001
Quaternary ammonium compound	75	225.1 ± 38.2	18.8 ± 6.6	92.0	< 0.001
Electronic-safe wipes	75	220.3 ± 42.5	40.1 ± 10.2	81.8	< 0.01

CFU: colony-forming units; SD: standard deviation. Reduction (%) = [(CFU before − CFU after)/CFU before] × 100. †Paired t-test comparing pre- and post-disinfection CFU within each group.

### IPC practices and MDR risk

Hand hygiene compliance was 62.0% before and 70.0% after patient contact. Daily phone disinfection was reported by only 9.0% and demonstrated the strongest protective association (22.2% vs. 63.4%; RR 0.35; 95% CI 0.17–0.71; *p* < 0.001). Awareness of IPC policies alone was not significantly protective (RR 1.08; *p* = 0.529), underscoring the primacy of behavioural implementation over knowledge alone (Table S4).

### Ward-level MDR prevalence and HAI rates

Ward-level MDR prevalence ranged from 40.0% (general ward) to 75.0% (ICU), paralleled by HAI rates varying from 6.1 to 14.8 per 1,000 patient-days. A strong positive association was observed (*r* = 0.986; 95% CI 0.84–0.99; *p* < 0.001), with MDR prevalence statistically accounting for 97% of the variability in HAI rates (R² = 0.97). For every 1% increase in MDR prevalence, the HAI rate increased by 0.26 cases per 1,000 patient-days (β = 0.26; SE = 0.02; *p* < 0.001). As this is an aggregate, ward-level analysis based on eight clinical areas and not adjusted for patient-level confounders such as acuity, antimicrobial use, or device density, it is interpreted as an exploratory finding. (Table 7).

**Table 7:** Ward-level multidrug-resistant prevalence and healthcare-associated infection rates (*n* = 8 wards).

Ward		MDR Prevalence (%)			HAI Rate (per 1,000 patient-days)
Intensive care unit		75			14.8
Burn unit		70			13.7
Surgical ward		61.8			12.9
Haematology/Oncology		57.1			11.2
Paediatric ward		52.5			9.4
Medical ward		46.7			8.1
Obstetrics/Gynaecology		45			7
General ward		40			6.1

Pearson r	95% CI	R²	Adjusted R²	β (SE)	p-value
Correlation and linear regression of MDR prevalence with HAI rate:					
0.986	0.84–0.99	0.97	0.97	0.26 (0.02)	< 0.001

HAI: healthcare-associated infection; CI: confidence interval; SE: standard error. β: increase in HAI rate (per 1,000 patient-days) per 1% increase in MDR prevalence; R²: coefficient of determination. Pearson correlation and simple linear regression; p ≤ 0.05 statistically significant.

### Phone behaviours and MDR contamination

Phone type (keypad vs. touchscreen) was not significantly associated with MDR prevalence (*p* = 0.660). In contrast, phone use during invasive procedures (72.4% vs. 50.3%; *p* < 0.001), restroom phone use (73.9% vs. 47.5%; *p* < 0.001), and phone use in critical-care areas (65.0% vs. 49.0%; *p* = 0.008) were all significantly associated with MDR. Daily disinfection was strongly protective (22.2% vs. 63.4%; *p* < 0.001) (Table S5).

### Independent predictors of MDR—multivariable analysis

Multivariable logistic regression identified four independent predictors. ICU/critical-care work was associated with more than a twofold increase in MDR odds (adjusted odds ratio [AOR] 2.4; 95% CI 1.4–4.2; *p* = 0.003). Phone use during invasive procedures was the strongest risk factor (AOR 2.9; 95% CI 1.7–5.0; *p* < 0.001). Restroom phone use was independently associated with increased risk (AOR 2.2; 95% CI 1.3–3.8; *p* = 0.006). Daily phone disinfection was independently protective, reducing MDR odds by approximately two-thirds (AOR 0.33; 95% CI 0.16–0.66; *p* < 0.001) (Table 8).

**Table 8:** Independent predictors of multidrug-resistant phone contamination by multivariable logistic regression (*n* = 300).

Variable	Adjusted OR (AOR)	95% Confidence Interval	*p*-value
Independent risk factors			
Working in ICU/critical-care setting	2.4	1.4–4.2	0.003
Phone use during invasive procedures	2.9	1.7–5.0	< 0.001
Phone use inside restroom	2.2	1.3–3.8	0.006
Independent protective factor			
Daily phone disinfection	0.33	0.16–0.66	< 0.001

Outcome: MDR contamination (yes/no). Variables were entered based on biological plausibility and univariate significance (p ≤ 0.05). AOR: adjusted odds ratio (OR < 1 indicates a protective effect); ICU: intensive care unit. p ≤ 0.05 statistically significant.

### Post-intervention follow-up—paired analysis

At two-week follow-up, overall bacterial contamination declined from 90.0% to 78.0% (*p* < 0.001), MDR contamination from 59.6% to 45.0% (*p* < 0.001), mean CFU from 190.1 ± 80.3 to 95.5 ± 55.2 (*p* < 0.001), and the proportion of high-contamination phones from 21.7% to 10.0% (*p* < 0.001) (Table 9).

**Table 9:** Post-intervention follow-up: paired comparison of contamination indicators at baseline versus two weeks (*n* = 300).

Outcome	Baseline	2-Week Follow-up	Statistical Test	*p*-value
Any bacterial contamination, n (%)	270 (90.0)	234 (78.0)	McNemar test	< 0.001
MDR contamination, n (%)	179 (59.6)	135 (45.0)	McNemar test	< 0.001
Mean CFU ± SD	190.1 ± 80.3	95.5 ± 55.2	Paired t-test	< 0.001
High CFU (> 200), n (%)	65 (21.7)	30 (10.0)	McNemar test	< 0.001

CFU: colony-forming units; MDR: multidrug resistance; SD: standard deviation. McNemar test for paired categorical variables; paired t-test for continuous variables. p ≤ 0.05 statistically significant.

## Discussion

The high contamination rate observed here supports the role of HCWs’ mobile phones as high-frequency fomites: frequent handling, infrequent disinfection, and repeated hand-and-face contact facilitate the acquisition, persistence, and transfer of skin- and environment-derived microorganisms. Comparable rates in Libya, Mexico, and Egypt indicate that this contamination is widespread rather than setting-specific^1,2,4,19–21^. The wide range reported across studies (10–100%) likely reflects differences in hand-hygiene compliance, disinfection practices, clinical setting, sampling, transport, and microbiological methods rather than true biological differences^4,6,22–25^. Together, these findings reinforce the importance of mobile phones as reservoirs of clinically important bacteria, with implications for healthcare-associated infections^3,6,8^.

The predominance of coagulase-negative *Staphylococci* (29.4%) and *S. aureus* (25.4%) is consistent with a mainly skin origin, as both colonize the hands and face and persist on dry surfaces^26^. Gram-negative organisms were less frequent but carried the greatest multidrug-resistance burden. This is consistent with their intrinsic and acquired resistance mechanisms — reduced membrane permeability, multidrug efflux, β-lactamase production, and mobile genetic elements — which enhance survival under antimicrobial pressure in *Acinetobacter baumannii*, *P. aeruginosa*, and *K. pneumoniae*^27–29^.

The high prevalence of MDR isolates likely reflects sustained antimicrobial selection pressure and intensive patient contact. Differences in stewardship, infection-prevention practices, and local resistance epidemiology probably explain why MDR prevalence here exceeded that reported by Tusabe et al. and Elgabeery et al.^1,21^ but was lower than that of Bodena et al.^24^.

The observed MRSA prevalence was broadly comparable to Sharma et al.^30^ and Loyola et al.^31^, lower than Selim and Abaza^32^ and Elgabeery et al.^21^, and higher than Pathare et al.^33^ and Galazzi et al.^34^. These differences likely reflect variation in local MRSA endemicity, infection-control practices, patient populations, and antimicrobial use. A recent study from our institution likewise documented a substantial MRSA burden in intensive care units^35^.

These ward-level differences were most marked in the ICU, which showed the highest contamination rate (98.3%), MDR prevalence (75.0%), and proportion of heavy contamination (> 200 CFU, 48.3%); ICU exposure was associated with markedly increased odds of MDR contamination (crude OR 4.5; *p* = 0.004). This is consistent with the high antimicrobial pressure, frequent invasive procedures, prolonged patient contact, and dense high-touch surfaces of critical-care environments, which facilitate the persistence and dissemination of resistant organisms^3,7,8,36^.

The susceptibility profiles revealed both preserved and compromised options. The absence of vancomycin resistance and very low linezolid resistance (2–3%) among Gram-positive isolates suggests these agents remain reliable for severe *Staphylococcal* infections, likely reflecting their restricted use. By contrast, the high penicillin and oxacillin resistance among *Staphylococci* reflects the spread of methicillin-resistant clones. Carbapenem resistance was most pronounced in *A. baumannii* (imipenem 30%; meropenem 32%) and *P. aeruginosa* (25–26%), reflecting intrinsic permeability barriers, efflux systems, and carbapenemase acquisition. TMP-SMX resistance exceeding 60% further limits options for these non-fermenting pathogens^3,37,38^.

Phenotypic ESBL production (24.9%) exceeded rates from Addis Ababa (8.3%)^39^ but was lower than those from Nepal (29.85%)^30^, Peru (33.3%)^31^, and Mexico (28%)^20^. Phenotypic carbapenemase production (13.0%) was comparable to Bodena et al.^24^ but lower than reports from India and Ghana^40,41^. The lower molecular detection rate (7.1%) than phenotypic positivity likely reflects the restricted PCR panel, which targeted only five carbapenemase genes (blaOXA-48, blaNDM, blaIMP, blaVIM, and blaKPC). Untested variants or non-enzymatic mechanisms — porin loss, efflux upregulation, or AmpC hyperproduction — may also contribute and warrant confirmation using broader molecular approaches.

Perfect agreement between phenotypic ESBL detection and PCR (κ = 1.00; *p* < 0.001) supports the reliability of phenotypic ESBL screening, whereas the weaker agreement for carbapenemases (κ = 0.67; *p* < 0.001) underscores the value of combining phenotypic and molecular methods for surveillance^13,16^. The absence of blaVIM and blaKPC, with blaOXA-48 as the predominant determinant, is consistent with previous reports from Mexico and Peru^20,31^.

All evaluated disinfectants significantly reduced contamination: sodium hypochlorite wipes achieved the greatest reduction (96.5%), followed by 70% isopropyl alcohol (94.8%), quaternary ammonium compound (92.0%), and electronic-safe wipes (81.8%), consistent with previous studies^10,21,42,43^. However, effectiveness depends on correct application, including adequate contact time and frequency. Although hypochlorite was most effective, its corrosive potential may damage device surfaces; alcohol-based or manufacturer-approved wipes are therefore more practical for routine use, with hypochlorite reserved for high-risk settings or outbreaks^44^.

Behavioural findings reinforced the importance of routine phone hygiene. Daily disinfection, reported by only 9.0% of HCWs, was independently protective (AOR 0.33; *p* < 0.001), whereas phone use during invasive procedures (AOR 2.9) and in restrooms (AOR 2.2) independently increased risk^45^. Notably, awareness of infection-prevention policies alone was not protective, indicating that effective interventions should prioritize behavioural change — through accessible disinfectants, structured education, and regular auditing — rather than knowledge alone.

At the institutional level, ward-level MDR prevalence on phones was strongly correlated with hospital-acquired infection (HAI) rates (*r* = 0.986; *p* < 0.001). This analysis was ecological, using ward-level aggregated data, and should be regarded as hypothesis-generating rather than confirmatory. Although the coefficient was exceptionally high, the small number of wards and the use of aggregate data preclude causal inference, and shared factors — ward acuity, antimicrobial pressure, patient vulnerability, and infection-prevention practices — may influence both MDR contamination and HAI rates. This exploratory association therefore warrants confirmation in larger multicentre studies using patient-level data. Nevertheless, the close alignment between microbial burden, MDR prevalence, and HAI rates, together with the post-intervention reduction in contamination, supports the hypothesis that mobile phones may contribute to the transmission ecology of healthcare-associated pathogens and reinforces their inclusion in infection-prevention strategies^3,5,8,14,46^.

Finally, the sustained reduction two weeks after a brief educational intervention shows that mobile-phone hygiene is readily modifiable. Integrating routine phone disinfection into existing IPC programmes — supported by evidence-based disinfectants, staff education, auditing, and feedback — offers a practical, scalable strategy to reduce contamination and strengthen antimicrobial-resistance control^9,14,38,42,47,48^.

## Conclusions

This study demonstrates a substantial burden of bacterial contamination and MDR on HCWs’ mobile phones, particularly in critical-care settings. The predominance of clinically significant pathogens and molecularly characterized resistance determinants supports the hypothesis that mobile phones may act as reservoirs of MDR organisms in the hospital environment. The strong ward-level association between MDR prevalence and HAI rates, though derived from aggregate data, underscores the epidemiological relevance of device-related contamination, and is further supported by the marked reduction in contamination achieved after a brief, targeted intervention. Routine, standardized mobile phone disinfection, integrated into IPC and antimicrobial stewardship programs, represents a practical, low-cost strategy to address this overlooked reservoir of resistant organisms.

## Limitations

Although the study included a prospective before-and-after intervention, the baseline observational component was cross-sectional, limiting causal inference. Microbial recovery may have been influenced by sampling conditions, and the discordance between phenotypic and molecular resistance detection may reflect untested resistance mechanisms or the limited molecular panel employed. The absence of molecular strain typing precluded assessment of clonal relatedness and direct transmission between mobile phones, patients, healthcare workers, and the hospital environment. The single-centre design, voluntary participation, and reliance on self-reported behaviours may have introduced selection, recall, and social-desirability biases, while the observed post-intervention improvements may have been influenced in part by the Hawthorne effect. In addition, the ward-level correlation between multidrug-resistant contamination and hospital-acquired infection rates represents an ecological, hypothesis-generating finding and should not be interpreted as evidence of causality. Finally, the long-term sustainability of the intervention was not evaluated, and although sodium hypochlorite was the most effective disinfectant, its potential to damage mobile devices may limit its routine use.

## Future perspectives

Future studies should integrate whole-genome sequencing and longitudinal designs to resolve transmission dynamics and resistance co-selection pathways. In parallel, novel sequence-specific antimicrobial strategies, including CRISPR-Cas-based approaches, warrant exploration as complementary tools against multidrug-resistant organisms^49^. Large-scale interventional trials are needed to determine whether sustained mobile phone hygiene can reduce MDR burden and HAI rates. Embedding standardized device disinfection within IPC frameworks may offer a scalable strategy to mitigate device-mediated transmission in healthcare settings.

## Supplementary Information

Below is the link to the electronic supplementary material.

Supplementary Material 1

Supplementary Material 2

Supplementary Material 3

## Data Availability

Datasets are available from the corresponding author upon reasonable request.
