# Supplementary material for: Multidrug-resistant bacterial contamination of healthcare workers’ mobile phones, decontamination efficacy, and potential implications for hospital-acquired infections
Source: Sci Rep. 2026 Aug 8;16:24547. doi: 10.1038/s41598-026-64278-1 (PMC13452803; doi:10.1038/s41598-026-64278-1)
Supplement: Supplementary file 1 [file 41598_2026_64278_MOESM3_ESM.docx]

**Supplementary Tables**

*Healthcare Workers’ Mobile Phones as Reservoirs of Multidrug-Resistant Bacteria in Intensive Care and High-Risk Hospital Settings*

**Table S1. Primer sequences used for multiplex PCR detection of extended-spectrum β-lactamase and carbapenemase genes.**

| **Gene** | **Primer Sequence (5′–3′)** | **Product (bp)** | **Ref.** |
| --- | --- | --- | --- |
| *blaTEM* | F: GAGTATTCAACATTTCCGTGTC / R: TAATCAGTGAGGCACCTATCTC | 800 | [17] |
| *blaSHV* | F: CGCCTGTGTATTATCTCCCTGTTAGCC / R: TTGCCAGTGCTCGATCAGCG | 843 | [17] |
| *blaCTX-M* | F: CGCTTTGCGATGTGCAG / R: ACCGCGATATCGTTGGT | 550 | [17] |
| *blaIMP* | F: GGAATAGAGTGGCTTAAYTCTC / R: GGTTTAAYAAAACAACCACC | 232 | [17] |
| *blaVIM* | F: GATGGTGTTTGGTCGCATA / R: CGAATGCGCAGCACCAG | 390 | [17] |
| *blaNDM* | F: GGTTTGGCGATCTGGTTTTC / R: CGGAATGGCTCATCACGATC | 621 | [17] |
| *blaKPC* | F: CGTCTAGTTCTGCTGTCTTG / R: CTTGTCATCCTTGTTAGGCG | 798 | [17] |
| *blaOXA-48* | F: GCGTGGTTAAGGATGAACAC / R: CATCAAGTTCAACCCAACCG | 438 | [17] |

*PCR: polymerase chain reaction. F: forward primer; R: reverse primer; bp: base pairs. Degenerate bases follow IUPAC nomenclature (Y = C/T; R = A/G; K = G/T; S = C/G). All primer sets were adopted from previously validated multiplex protocols [17].*

**Table S2. Antimicrobial resistance rates (%) among major bacterial isolates.**

| **Antimicrobial Agent** | ***S. aureus* (n = 95)** | **CoNS (n = 110)** | ***K. pneumoniae* (n = 60)** | ***E. coli* (n = 45)** | ***P. aeruginosa* (n = 35)** | ***A. baumannii* (n = 29)** |
| --- | --- | --- | --- | --- | --- | --- |
| Penicillin | 95 | 90 | — | — | — | — |
| Oxacillin | 58 | 65 | — | — | — | — |
| Erythromycin | 42 | 50 | — | — | — | — |
| Clindamycin | 28 | 35 | — | — | — | — |
| TMP-SMX | 25 | 30 | 38 | 36 | 60 | 62 |
| Vancomycin | 0 | 0 | — | — | — | — |
| Linezolid | 2 | 3 | — | — | — | — |
| Daptomycin | 0 | 0 | — | — | — | — |
| Amoxicillin–clavulanate | — | — | 48 | 44 | — | — |
| Piperacillin–tazobactam | — | — | 34 | 30 | 40 | 55 |
| Ceftazidime | — | — | 42 | 41 | 55 | 58 |
| Cefepime | — | — | 38 | 35 | 50 | 54 |
| Aztreonam | — | — | 36 | 34 | 48 | — |
| Ciprofloxacin | 15 | 22 | 35 | 33 | 45 | 50 |
| Tetracycline | 30 | 40 | 42 | 40 | 60 | 55 |
| Gentamicin | 18 | 28 | 31 | 30 | 50 | 52 |
| Imipenem | — | — | 15 | 13 | 25 | 30 |
| Meropenem | — | — | 18 | 15 | 26 | 32 |

*Values represent the percentage of resistant isolates. —: not applicable / not tested. CoNS: coagulase-negative staphylococci; TMP-SMX: trimethoprim–sulfamethoxazole. Resistance was interpreted per CLSI 2025 criteria.*

**Table S3. Species-level resistance gene distribution and phenotype–genotype diagnostic performance (n = 169).**

**A. Species-level gene distribution**

| **Species** | **Phenotypic ESBL (n)** | ***blaTEM* n (%)** | ***blaSHV* n (%)** | ***blaCTX-M* n (%)** | **Phenotypic Carb. (n)** | ***blaOXA-48* n (%)** | ***blaNDM* n (%)** | ***blaIMP* n (%)** | ***blaVIM* / *blaKPC*** |
| --- | --- | --- | --- | --- | --- | --- | --- | --- | --- |
| *K. pneumoniae* | 11 | 6 (54.5) | 3 (27.3) | 2 (18.2) | 2 | 1 (50.0) | 0 | 0 | 0 / 0 |
| *E. coli* | 16 | 9 (56.3) | 4 (25.0) | 3 (18.8) | 4 | 1 (25.0) | 1 (25.0) | 0 | 0 / 0 |
| *P. aeruginosa* | 8 | 4 (50.0) | 2 (25.0) | 2 (25.0) | 7 | 2 (28.6) | 1 (14.3) | 0 | 0 / 0 |
| *A. baumannii* | 7 | 4 (57.1) | 3 (42.9) | 1 (14.3) | 9 | 4 (44.4) | 1 (11.1) | 1 (11.1) | 0 / 0 |
| **Total** | **42** | **23 (54.8)** | **12 (28.6)** | **8 (19.0)** | **22** | **8 (36.4)** | **3 (13.6)** | **1 (4.5)** | **0 / 0** |

**B. Diagnostic performance of phenotypic versus molecular detection**

| **Resistance Mechanism** | **Sensitivity (%)** | **Specificity (%)** | **PPV (%)** | **NPV (%)** | **Diagnostic Accuracy (%)** | **Cohen’s κ** | **p-value** |
| --- | --- | --- | --- | --- | --- | --- | --- |
| ESBL | 100 | 100 | 100 | 100 | 100 | 1.00 | < 0.001 |
| Carbapenemase | 54.5 | 100 | 100 | 93.6 | 94.1 | 0.67 | < 0.001 |

*ESBL: extended-spectrum β-lactamase; Carb.: carbapenemase; PPV: positive predictive value; NPV: negative predictive value; κ: Cohen’s kappa (phenotypic–molecular agreement). Gene percentages (Part A) are calculated within phenotypically positive isolates; co-carriage may cause gene counts to exceed isolate counts.*

**Table S4. Infection-prevention-and-control compliance practices and association with multidrug-resistant contamination risk (n = 300).**

| **IPC Variable** | **Compliant n (%)** | **MDR: Compliant n (%)** | **MDR: Non-compliant n (%)** | **Risk Diff. (%)** | **RR (95% CI)** | **p-value** |
| --- | --- | --- | --- | --- | --- | --- |
| Hand hygiene before patient contact | 186 (62.0) | 90 (48.4) | 89 (78.1) | −29.7 | 0.62 (0.52–0.74) | < 0.001 |
| Hand hygiene after patient contact | 210 (70.0) | 92 (43.8) | 87 (96.7) | −52.9 | 0.45 (0.39–0.53) | < 0.001 |
| Consistent glove use during procedures | 228 (76.0) | 120 (52.6) | 59 (81.9) | −29.3 | 0.64 (0.55–0.76) | < 0.001 |
| Daily phone disinfection | 27 (9.0) | 6 (22.2) | 173 (63.4) | −41.1 | 0.35 (0.17–0.71) | < 0.001 |
| Weekly workstation disinfection | 138 (46.0) | 65 (47.1) | 114 (70.4) | −23.3 | 0.67 (0.55–0.82) | < 0.001 |
| Avoiding phone use in high-risk areas | 94 (31.3) | 40 (42.6) | 139 (67.5) | −24.9 | 0.63 (0.49–0.81) | < 0.001 |
| Formal IPC training (≤ 2 years) | 160 (53.3) | 72 (45.0) | 107 (76.4) | −31.4 | 0.59 (0.48–0.72) | < 0.001 |
| Awareness of IPC policy (knowledge only) | 248 (82.7) | 150 (60.5) | 29 (55.8) | +4.7 | 1.08 (0.83–1.41) | 0.529 |
| Proper phone storage outside sterile field | 172 (57.3) | 80 (46.5) | 99 (77.3) | −30.8 | 0.60 (0.50–0.72) | < 0.001 |

*IPC: infection prevention and control; MDR: multidrug resistance; RR: risk ratio; CI: confidence interval. Risk difference: absolute difference in MDR prevalence between compliant and non-compliant groups. p-values by Chi-square test (Fisher’s exact test when expected cell counts < 5).*

**Table S5. Association between phone characteristics and behaviours and multidrug-resistant contamination (n = 300).**

| **Variable** | **Category** | **MDR (n = 179) n (%)** | **Non-MDR (n = 121) n (%)** | **Total (n)** | **χ²** | **p-value** |
| --- | --- | --- | --- | --- | --- | --- |
| **Phone type** | Touchscreen only | 168 (60.0) | 112 (40.0) | 280 | 0.19 | 0.660 |
|  | Keypad/buttons | 11 (55.0) | 9 (45.0) | 20 |  |  |
| **Use in critical-care areas** | Yes | 130 (65.0) | 70 (35.0) | 200 | 7.09 | 0.008 |
|  | No | 49 (49.0) | 51 (51.0) | 100 |  |  |
| **Answering calls during patient care** | Yes | 115 (66.1) | 59 (33.9) | 174 | 7.11 | 0.008 |
|  | No | 64 (50.8) | 62 (49.2) | 126 |  |  |
| **Phone use during invasive procedures** | Yes | 92 (72.4) | 35 (27.6) | 127 | 14.93 | < 0.001 |
|  | No | 87 (50.3) | 86 (49.7) | 173 |  |  |
| **Sharing phone with colleagues** | Yes | 104 (61.5) | 65 (38.5) | 169 | 0.56 | 0.453 |
|  | No | 75 (57.3) | 56 (42.7) | 131 |  |  |
| **Phone use inside restroom** | Yes | 102 (73.9) | 36 (26.1) | 138 | 21.55 | < 0.001 |
|  | No | 77 (47.5) | 85 (52.5) | 162 |  |  |
| **Daily phone disinfection** | Yes | 6 (22.2) | 21 (77.8) | 27 | 17.29 | < 0.001 |
|  | No | 173 (63.4) | 100 (36.6) | 273 |  |  |

*MDR: multidrug resistance. Percentages are row-wise. Associations were assessed by Chi-square test.* p ≤ 0.05 statistically significant.
